# A novel serogenetic approach determines the community prevalence of celiac disease and informs improved diagnostic pathways

**DOI:** 10.1186/1741-7015-11-188

**Published:** 2013-08-28

**Authors:** Robert P Anderson, Margaret J Henry, Roberta Taylor, Emma L Duncan, Patrick Danoy, Marylia J Costa, Kathryn Addison, Jason A Tye-Din, Mark A Kotowicz, Ross E Knight, Wendy Pollock, Geoffrey C Nicholson, Ban-Hock Toh, Matthew A Brown, Julie A Pasco

**Affiliations:** 1 The Walter and Eliza Hall Institute of Medical Research, 1G Royal Parade, Parkville, Victoria 3052, Australia; 2 Department of Medical Biology, The University of Melbourne, Parkville, Victoria 3010, Australia; 3 Department of Gastroenterology, The Royal Melbourne Hospital, Melbourne Health, Grattan St, Parkville, Victoria 3050, Australia; 4 Current address: ImmusanT, Inc., ImmusanT Inc., One Kendall Square, Building 200, LL, Suite 4, Cambridge, MA 02139, USA; 5 School of Medicine, Deakin University, Geelong, Victoria, Australia; 6 Healthscope Pathology, Melbourne, Victoria, Australia; 7 Human Genetics Group, University of Queensland Diamantina Institute, Level 5, Translational Research Institute, 37 Kent St, Woolloongabba, QLD 4102, Australia; 8 Endocrinology, Royal Brisbane and Women’s Hospital, Butterfield Rd, Herston, QLD 4029, Australia; 9 NorthWest Academic Centre, Department of Medicine, The University of Melbourne, St Albans, Victoria, Australia; 10 Geelong Gastroenterology, Level 1, 83 Myers St, Geelong, Victoria 3220, Australia; 11 Rural Clinical School, School of Medicine, The University of Queensland, Toowoomba, QLD 4350, Australia; 12 Current address: Roche Diagnostics Australia, 31 Victoria Avenue, Castle Hill, New South Wales 2154, Australia

**Keywords:** Celiac disease, Diagnosis, Epidemiology, Serology, Transglutaminase, Deamidated gliadin peptide, Immunogenetics, Prevalence

## Abstract

**Background:**

Changing perspectives on the natural history of celiac disease (CD), new serology and genetic tests, and amended histological criteria for diagnosis cast doubt on past prevalence estimates for CD. We set out to establish a more accurate prevalence estimate for CD using a novel serogenetic approach.

**Methods:**

The human leukocyte antigen (HLA)-DQ genotype was determined in 356 patients with ‘biopsy-confirmed’ CD, and in two age-stratified, randomly selected community cohorts of 1,390 women and 1,158 men. Sera were screened for CD-specific serology.

**Results:**

Only five ‘biopsy-confirmed’ patients with CD did not possess the susceptibility alleles HLA-DQ2.5, DQ8, or DQ2.2, and four of these were misdiagnoses. HLA-DQ2.5, DQ8, or DQ2.2 was present in 56% of all women and men in the community cohorts. Transglutaminase (TG)-2 IgA and composite TG2/deamidated gliadin peptide (DGP) IgA/IgG were abnormal in 4.6% and 5.6%, respectively, of the community women and 6.9% and 6.9%, respectively, of the community men, but in the screen-positive group, only 71% and 75%, respectively, of women and 65% and 63%, respectively, of men possessed HLA-DQ2.5, DQ8, or DQ2.2. Medical review was possible for 41% of seropositive women and 50% of seropositive men, and led to biopsy-confirmed CD in 10 women (0.7%) and 6 men (0.5%), but based on relative risk for HLA-DQ2.5, DQ8, or DQ2.2 in all TG2 IgA or TG2/DGP IgA/IgG screen-positive subjects, CD affected 1.3% or 1.9%, respectively, of females and 1.3% or 1.2%, respectively, of men. Serogenetic data from these community cohorts indicated that testing screen positives for HLA-DQ, or carrying out HLA-DQ and further serology, could have reduced unnecessary gastroscopies due to false-positive serology by at least 40% and by over 70%, respectively.

**Conclusions:**

Screening with TG2 IgA serology and requiring biopsy confirmation caused the community prevalence of CD to be substantially underestimated. Testing for HLA-DQ genes and confirmatory serology could reduce the numbers of unnecessary gastroscopies.

## Background

It is not uncommon for patients suspected of having celiac disease (CD) to be reluctant to undergo definitive testing by small bowel biopsy. By contrast, an online community survey of randomly selected Australians indicated that 28% of adults monitor their dietary gluten intake, with 4% avoiding it altogether, 5% strictly controlling intake, and a further 18% loosely controlling consumption (Coeliac Australia, personal communication). Despite the current popularity of gluten-free food, it is unclear what proportion of the community with CD is diagnosed or remains unrecognized [1,2].

Over the past 20 years or more, widely implemented expert guidelines have recommended that diagnosis of CD should be based on small bowel histology showing villous atrophy and confirmed by symptoms, laboratory abnormalities, and/or intestinal histology showing improvement with exclusion of dietary gluten [3]. However, several developments have challenged this definition. Recently the European Society for Pediatric Gastroenterology, Hepatology, and Nutrition (ESPGHAN) Working Group on Coeliac Disease Diagnosis proposed that CD should be redefined as ‘an immune-mediated systemic disorder elicited by gluten and related prolamines in genetically susceptible individuals, characterized by the presence of a variable combination of gluten-dependent clinical manifestations, CD-specific antibodies, human leukocyte antigen (HLA)-DQ2 and HLA-DQ8 haplotypes, and enteropathy’ [4]. The ESPGHAN Working Group also concluded CD is frequently difficult to recognize ‘because of the variation in presentation and intensity of symptoms and signs, and many cases may actually occur without symptoms’ [4]. Consequently, a new approach to the diagnosis of CD has recently been published, in which for the first time, HLA-DQ genotyping was included as the initial step in testing for CD in patients who are considered at risk of CD, but do not have the typical symptoms. In many countries with sophisticated healthcare such as the USA and most of Europe, by far the majority of patients with CD in the community remain undiagnosed and do not present to their physicians with the so-called ‘typical’ digestive symptoms. However, patients presenting in primary care and in many sub-specialty clinics frequently have symptoms and co-morbidities that raise the possibility of CD, or might have adopted a gluten-free diet without prior assessment for CD [5]. Even if physicians were more aware of the diverse clinical presentations of CD and the availability of new serological and genetic tests, it is not yet clear how to efficiently and cost-effectively assess the possibility of CD in patients without typical gastrointestinal symptoms.

Almost all credible community prevalence studies for CD have relied on sera being initially screened by ‘CD-specific serology’, followed by additional supportive serology or ‘definitive’ intestinal histology showing villous atrophy [2]. However, the frequencies of false-positive and false-negative IgA endomysial immunofluorescence (EMA) and tissue transglutaminase (TG)-2 serology assays can vary widely. In part, this uncertainty arises because abnormal intestinal histology remains the gold standard for diagnosis. In community prevalence studies, there is the practical problem that many subjects are unable or refuse to have gastroscopies to allow intestinal histology to be ascertained. A further issue is what constitutes abnormal histology compatible with CD, particularly if only mild inflammation showing increased density of intra-epithelial lymphocytes (IELs) is found without evidence of villous atrophy [1,6-10].

The first CD prevalence studies from Australia highlight these challenges [11,12]. Based on a single set of sera collected 5 years earlier, 10 of 3,011 adults were found to be positive for EMA, and all 7 who underwent gastroscopy were confirmed by intestinal histology as having CD, thus when 2 cases already diagnosed based on biopsy were combined with those found by screening, the estimated prevalence of CD was 0.4%. In a follow-up study 5 years later, using the same serum set, TG2 IgA or IgG serology was found to be raised in 47 sera, and CD was confirmed after intestinal histology in 14 of 31 seropositive subjects available for follow-up [11]. Thus, the investigators offered a revised prevalence estimate of at least 0.56% based on histology-confirmed cases, but proposed that the rate of affected subjects could be as high as 0.96% if other seropositive subjects who carried HLA-DQ2 or DQ8 were also included.

An outstanding feature of CD is that almost all affected individuals possess immune recognition genes encoding either HLA-DQ2.5, DQ8, or DQ2.2, which facilitate CD4 T-cell recognition of specific gluten-derived peptides [13-17]. HLA typing for DQ2.5, DQ8, and/or DQ2.2 is an exceptionally powerful negative predictor, but is of little value as a positive predictor of CD in individual patients, because these susceptibility genes are found in 30 to 40% of the general community. Large-scale, high-throughput HLA-DQ genotyping is now inexpensive in the research setting, but remains surprisingly expensive in some healthcare systems when used in clinical practice.

In the current study, we tested the positive predictive power of HLA-DQ genotyping and serology to estimate the prevalence of CD at a population level. This novel approach has the potential to overcome the limitations imposed by the requirement for intestinal histology to assess population prevalence accurately. Our findings also led us to develop a series of diagnostic algorithms to compare costs and resource utilization, and these could be used to inform clinical decision-making when evaluating the possibility of CD in patients not considered at high risk.

## Methods

### Study populations

The community cohorts used in this study have been described previously [18,19]. Briefly, subjects were recruited to both female and the male age-stratified, randomly selected cohorts, based on the electoral rolls in the Barwon Statistical Division, a geographically well-defined, large, stable population of 259,013 people (2006 Australian Bureau of Statistics Catalogue number 2001.0) whose educational and socioeconomic characteristics and age structure differ by less than 5% from the national Australian profiles [19]. Participation rates were 77% for the female cohort and 65% for the male cohort. The female cohort was recruited between 1994 and 1997 (n = 1494; median age 54 years, range 20 to 94 years), and the male cohort was recruited between 2001 and 2006 (n = 1540; median age 56 years, range 20 to 97 years). Blood was taken from all participants at enrolment after an overnight fast. Sera that were stored frozen at −80°C for 13 to 16 years (females) or 4 to 9 years (males) were available for 1,390 women and 1,158 men. DNA extracted from whole blood and then stored at −80°C was also available for 1,177 of the women and 1,056 of the men who had provided sera. Frozen sera collected 10 years later were also available from 597 of the 1,390 subjects in the female cohort. Subjects in the female cohort were invited to return every 2 years, and those in the male cohort to return every 5 years. At enrolment and at each review, participants completed questionnaires about their demographics, general health, medication exposure, and disease histories. Medical history was derived from the self-reported history of diseases, and where possible validated by reference to the participants’ medical records. In 2010, volunteers whose de-identified data indicated abnormal composite TG2/deamidated gliadin peptide (DGP) IgG and IgA serology that was then confirmed by raised levels of TG2 IgA, DGP IgG (DGP-G), or DGP IgA (DGP-A) were contacted by letter and informed that their blood tests suggested CD, and that although this did not diagnose CD, they ought to see their doctor as soon as possible to discuss appropriate management.

In a separate study, volunteers aged between 18 and 70 years old with ‘biopsy-confirmed’ CD based on the 1990 ESPGHAN diagnostic criteria [3] were recruited at The Royal Melbourne Hospital following advertisements in the Victorian State Coeliac Society newsletter. HLA-DQ genotypes were established for these subjects, and these have been partially reported elsewhere [17].

### Ethics

Approval by the appropriate ethics committees was obtained by each of the participating centers. The Barwon Health Human Research and Ethics Committee granted approval for the original prospective community cohorts (92/01 and 00/56). An amendment in 2009 allowed testing for CD (92/01-s5 and 00/56-s5), and The Melbourne Health Human Research Ethics Committee approved recruitment of volunteers with biopsy-confirmed CD for the study ‘Gluten Immunity in Coeliac Disease’ (2003.009). Written informed consent was obtained from each volunteer participating in the study.

### Serology

A single clinical diagnostic immunology scientist (RT) performed all serological assays in a commercial pathology laboratory (Healthscope Pathology, Clayton, Victoria, Australia). Sera from 1,390 women (95% of the female cohort) and 1,158 men (98% of the male cohort) were thawed once, divided into smaller aliquots, and stored at −80°C before being transferred to the clinical laboratory. The thawed sera were tested in batches for anti-native human TG2 IgA (h-tTG IgA; QUANTA Lite®; product number 708760), anti-gliadin IgG (AGG; QUANTA Lite® Gliadin IgG; number 708650), anti-gliadin IgA (AGA; QUANTA Lite® Gliadin IgA; number 708655) (all INOVA Diagnostics Inc., San Diego, CA, USA), and in a composite antigen ELISA that measures IgA and IgG specific for native human red cell-derived TG2 and synthetic, deamidated gliadin-derived peptides (composite TG2/DGP IgA/IgG; QUANTA Lite® h-tTG/DGP Screen; number 704575; INOVA Diagnostics Inc.). If the result of the initial TG2 IgA ELISA was at or above the manufacturer’s cut-off value of 20 units, then a sample of the same aliquot of serum was also tested for EMA (monkey esophagus IFA Kit; binding site FK208.2/INOVA number I704150; INOVA Diagnostics Inc.). When EMA was negative and TG2 IgA abnormal, the same serum was tested using another commercial TG2 IgA ELISA kit (1910–9601 A; Euroimmun AG, Lubeck, Germany). If the result of the composite TG2/DGP IgA/IgG screen was at or above the manufacturer’s upper limit of normal (ULM), that is 20 U,) the same sample of serum was tested for TG2 IgA (QUANTA Lite® h-tTG IgA; number 708760), DGP-G (QUANTA Lite® Gliadin IgG II; number 704520) and DGP-A (QUANTA Lite® Gliadin IgA II; number 704525) (all INOVA Diagnostics Inc.)

### HLA-DQ genotyping

In the community cohort, DNA was extracted from the blood collected at enrolment. Five single-nucleotide polymorphisms (SNPs) (rs2187668, rs2395182, rs4713586, rs7454108, and rs7775228) in linkage disequilibrium with the CD-associated HLA risk factors DQ2.5, DQ8, and DQ2.2, which indicate direct risk, and with DQA1*0505/DQB1*0301 (DQ7), which contributes to the risk of DQ2.5 in CD when also inherited with DQA1*0201/DQB1*0202 (DQ2.2) were used to determine HLA-DQ haplotypes, as previously described [20,21]. Genotyping was performed by the Human Genetics Group (t the University of Queensland Diamantina Institute) using Taqman SNP genotyping methods (Applied Biosystems, Foster City, CA, USA) following the manufacturer’s protocols. Full SNP-based HLA-DQ genotyping was possible for 1,065 (90%) of the 1,177 DNA samples provided from the female cohort and 921 (87%) of the DNA samples from the male cohort; hence, complete HLA-DQ genotype and serology datasets were available for 73% of the total female cohort and 78% of the total male cohort. For subjects from the cohort of patients with biopsy-confirmed CD and for subjects in the community female and male cohorts with higher readings in the composite TG2/DGP IgA/IgG screen, which were subsequently confirmed by increased TG2 IgA, DGP-G or DGP-A levels, the HLA-DQ genotypes were also determined in a clinical immunogenetics laboratory using the PCR to sequence-specific oligonucleotide (SSO) hybridization method (Victorian Transplantation and Immunogenetics Service, Parkville, VIC, Australia) [22-24]. HLA-DQ2.5, DQ8, and DQ2.2 genotypes determined by both methods for 73 subjects were in agreement, except in 1 case. which was determined to be HLA-DQ2.5^+^DQ8^-^DQ2.2^-^ by SSO and HLA-DQ2.5^-^DQ8^-^2.2^+^ by SNP genotyping.

### Statistical analysis

The GraphPad Prism software package (GraphPad Software, Inc. La Jolla, CA, USA) was used for statistical calculations. Serological status and HLA-DQ genotype were compared by Fisher’s exact test. A probability of less than 5% by two-tailed analysis was taken to be significant.

For the 1,065 subjects from the female cohort, and 921 subjects from the male cohort with paired HLA-DQ genotype and serology data, the estimated number with CD (n) was calculated using contingency tables as the excess of subjects found to be positive for one or an indicated series of serological tests and also gene-positive for HLA-DQ2.5, DQ8, or DQ2.2 (a_*O*_) above those expected (a_*E*_), minus the difference between the observed (c_*O*_) and expected (c_*E*_) number of subjects who were seropositive and gene-negative for HLA-DQ2.5, DQ8, and DQ2.2:

(1) $$n=\left(a_{O-}-a_{E}\right)-\left(c_{O}-c_{E}\right)$$

here the expected number of seropositive and gene-positive subjects is:

(2) $$a_{E}=\left(a_{O-}+c_{O}\right)\times \left(a_{O-}+b_{O}\right)÷\left(a_{O-}+b_{O-}+c_{O-}+d_{O}\right)$$

where b_*O*_ is the observed number of seronegative and gene-positive subjects, and d_*O*_ is the observed number of seronegative and of gene-negative subjects. Similarly, the expected number of seropositive and of gene-negative subjects is given by:

(3) $$c_{E}=\left(a_{O-}+c_{O}\right)\times \left(c_{O-}+d_{O}\right)÷\left(a_{O-}+b_{O-}+c_{O-}+d_{O}\right)$$

The upper (n_*UL*_) and lower (n_*LL*_) 95% confidence intervals (CIs) for the estimated numbers of subjects with CD (n) were derived using equation 1, and the upper (RR_*UL*_) and lower (RR_*LL*_) 95% confidence intervals of the relative risk (RR) for being gene-positive in the seropositive subjects were calculated by GraphPad Prism, thus:

(4) $$\mathrm{RR}=\left(a_{O}÷\left(a_{O-}+c_{O}\right)\right)÷\left(b_{O-}÷\left(b_{O}+d_{O}\right)\right)$$

and therefore, the upper 95% CI for the number of subjects seropositive and gene-positive (a_*UL*_) is given by:

(5) $$a_{\mathrm{UL}}={\left(RR_{\mathrm{UL}}\times \left(a_{O}+c_{O}\right)\times b_{O}\right)}_{-}÷\left(b_{O}+d_{O}\right)$$

Similarly, the lower 95% CI (a_*LL*_) for the number of subjects seropositive and gene-positive is given by:

(6) $$a_{\mathrm{LL}}=\left(RR_{\mathrm{LL}}\times \left(a_{O}+c_{O}\right)\times b_{O}\right)÷\left(b_{O}+d_{O}\right)$$

The upper 95% CI for the number of subjects seropositive and gene-negative (c_*UL*_) is then:

(7) $$c_{\mathrm{UL}}=\left(a_{O}+c_{O}\right)-a_{\mathrm{UL}}$$

and similarly, the lower 95% CI for the number of subjects seropositive and gene-negative (c_*LL*_) is then:

(8) $$c_{\mathrm{LL}}=\left(a_{O}+c_{O}\right)-a_{\mathrm{LL}}$$

Thus, the upper 95% CI (n_*UL*_) for the number of subjects with CD is given by:

(9) $$n_{\mathrm{UL}}=\left(a_{\mathrm{UL}}-a_{E}\right)+\left(c_{E}-c_{\mathrm{UL}}\right)$$

and the lower 95% CI (n_*LL*_) for the number of cases with CD is then:

(10) $$n_{\mathrm{LL}}=\left(a_{\mathrm{LL}}-a_{E}\right)+\left(c_{E}-c_{\mathrm{LL}}\right)$$

### Costing of diagnostic algorithms

Australia has a publicly funded health system that provides primary healthcare treatment for all Australian citizens and permanent residents, and is operated by the government authority Medicare Australia. To estimate the cost of investigations incurred for a hypothetical cohort of 1,000 adult men or women in the community being tested for CD, the full Australian Medicare Benefits Schedule fees were applied (http://www9.health.gov.au/mbs/search.cfm). The cost code 71163 (A$1 = US$0.91; A$24.90) was applied for initial TG2 IgA estimation or composite TG2/DGP IgA/IgG screen; 71164 (A$40.15) for confirmatory EMA (combined with repeat TG2 IgA) or TG2 IgA, DGP-G, and DGP-G; 71151 (A$119.65) for HLA-DQ genotyping; and an aggregated estimate of A$850 for diagnostic gastroscopy performed in a community endoscopy center by a specialist gastroenterologist, with sedation provided by a medical practitioner sedationist, and standard histology of five biopsies collected from the proximal small intestine. The costs of follow-up appointments with the treating doctor, and personal costs borne by the patient associated with attendance for blood collection and gastroscopy were not included. For the purpose of modeling the effectiveness of the diagnostic algorithms, we assumed that small bowel histology correctly identified all patients with CD predicted by serogenetic prevalence estimates. However, it should be noted that small bowel biopsies collected from approximately 10% of patients are not suitable for definitive diagnosis, and inter-observer variability between histopathologists can be substantial [25,26]. Per capita claims to Medicare Australia for one of two item numbers dedicated to CD serology testing (71163 and 71164) were sourced from the website of Medicare Australia (https://www.medicareaustralia.gov.au/statistics/mbs_item.shtml).

## Results

### HLA-DQ status and biopsy-confirmed CD

We first determined the prevalence of HLA-DQ2.5, DQ8, or DQ2.2 in local members of the celiac support group who responded to an invitation for volunteers with ‘biopsy-proven’ CD. Of 356 patients with a diagnosis of CD confirmed by small bowel histology, HLA-DQ2.5 was present in 325 patients (91.3%), HLA-DQ8 but not HLA-DQ2.5 was found in 19 (5.3%), and DQ2.2 but not HLA-DQ2.5 or HLA-DQ8 was present in 7 (2.0%) (Table 1); only 5 patients (1.4%) lacked HLA-DQ2.5, HLA-DQ8 and HLA-DQ2.2. Clinical review led to CD being excluded in four of these ‘gene-negative’ cases because the small bowel histology remained normal after prolonged gluten challenge, and in two cases because the intestinal histology and further investigations led to a diagnosis of Crohn’s disease or common variable immunodeficiency. One gene-negative case declined clinical re-evaluation. Hence, at least 99.7% of local patients with histology-confirmed CD possessed HLA-DQ2.5, HLA-DQ8, or HLA-DQ2.2.

**Table 1:** Serology, HLA-DQ status, and inferred prevalence of CD

	**Total**	**HLA-DQ 2.5/8/2.2-positive, observed,(expected) n**	**HLA-DQ 2.5/8/2.2-positive, RR (95% CI)**	**Excess HLA-DQ 2.5/8/2.2-positive, n**	**Prevalence per 1.000 (95% CI)**
Biopsy-confirmed CD					
F + M	356	351	1.78 (1.71 to 1.84)^a^		NA
Biopsy-confirmed CD; four of five HLA-DQ2.5-DQ8-DQ2.2- re-investigated					
F + M	352	351	1.80 (1.73 to 1.89)^a^		NA
Community random sample of age-stratified cohorts of adults					
F	1,065	596	1		0
M	921	513	1		0
Community sample: initially positive for TG2 IgA					
F	48	34 (27)	1.28 (1.06 to 1.55)^b^	14	13.4 (2.4 to 26.8)
M	68	44 (38)	1.18 (0.98 to 1.42)^NS^	12	13.2 (−2.9 to 32.8)
Community sample: initially positive for TG2 IgA, and confirmed positive for EMA					
F	13	13 (7)	1.80 (1.71 to 1.90)^c^	11	10.8 (9.5 to 12.1)
M	13	13 (7)	1.82 (1.71 to 1.93)^c^	12	12.5 (10.9 to 14.2)
Community sample: initially positive for composite TG2/DGP IgA/IgG					
F	55	41 (31)	1.36 (1.15 to 1.60)^d^	20	19.2 (7.5 to 32.9)
M	73	46 (41)	1.14 (0.95 to 1.38) ^NS^	11	11.6 (−5.4 to 32.0)
Community sample: initially positive for composite TG2/DGP IgA/IgG, and confirmed positive for TG2 IgA, DGP-G-+, or DGP-A					
F	37	30 (21)	1.47 (1.23 to 1.74)^e^	19	17.5 (8.9 to 27.6)
M	44	29 (25)	1.19 (0.96 to 1.49) ^NS^	9	9.8 (−2.7 to 25.3)
Community sample: – initially positive for TG2/DGP IgA/IgG, and confirmed positive for TG2 IgA, DGP-G, and DGP-A					
F	10	10 (6)	1.80 (1.71 to 1.90)^d^	9	8.3 (7.3 to 9.3)
M	11	11 (6)	1.82 (1.72 to 1.93)^e^	10	10.6 (9.3 to 12.1)

Abbreviations: CD, celiac disease; DGP, Deamidated gliadin-derived peptide; EMA, endomysial immunofluorescence IgA; F, female; HLA, human leukocyte antigen; Ig, immunoglobulin; M, male; NA, not applicable; NS, not significant; RR, relative risk; TG2, transglutaminase-2.

Compared with community M + F: ^a^*P*<0.0001 (χ^2^ two-tailed); ^b^*P*<0.04, ^c^*P*<0.001, ^d^*P*<0.005, ^e^*P*<0.002, (Fisher’s exact test, two-tailed).

### Community prevalence of celiac disease HLA-DQ susceptibility genotypes

In the female and male cohorts recruited from the community, 55.9% and 55.7% of genotyped subjects, respectively, were genetically susceptible to CD because they were positive for HLA-DQ2.5, DQ8, or DQ2.2. The proportions of the genotyped women and men positive for HLA-DQ2.5 were virtually identical: 24.3% versus 24.7%, respectively. Subjects negative for HLA-DQ2.5 but positive for HLA-DQ8 accounted for 15.7% of women and 16.1% of men; and subjects who were negative for both HLA-DQ2.5 and HLA-DQ8 but positive for HLA-DQ2.2 represented 16.0% of women and 15.0% of men. Accordingly, the RR for HLA-DQ2.5, DQ8, or DQ2.2 in patients with CD compared with community subjects was 1.80 (95% CI 1.71 to 1.90) for women and 1.82 (95% CI 1.71 193) for men (Table 1).

### TG2 IgA serology and genetic evidence of celiac disease in the community

**Figure 1:**
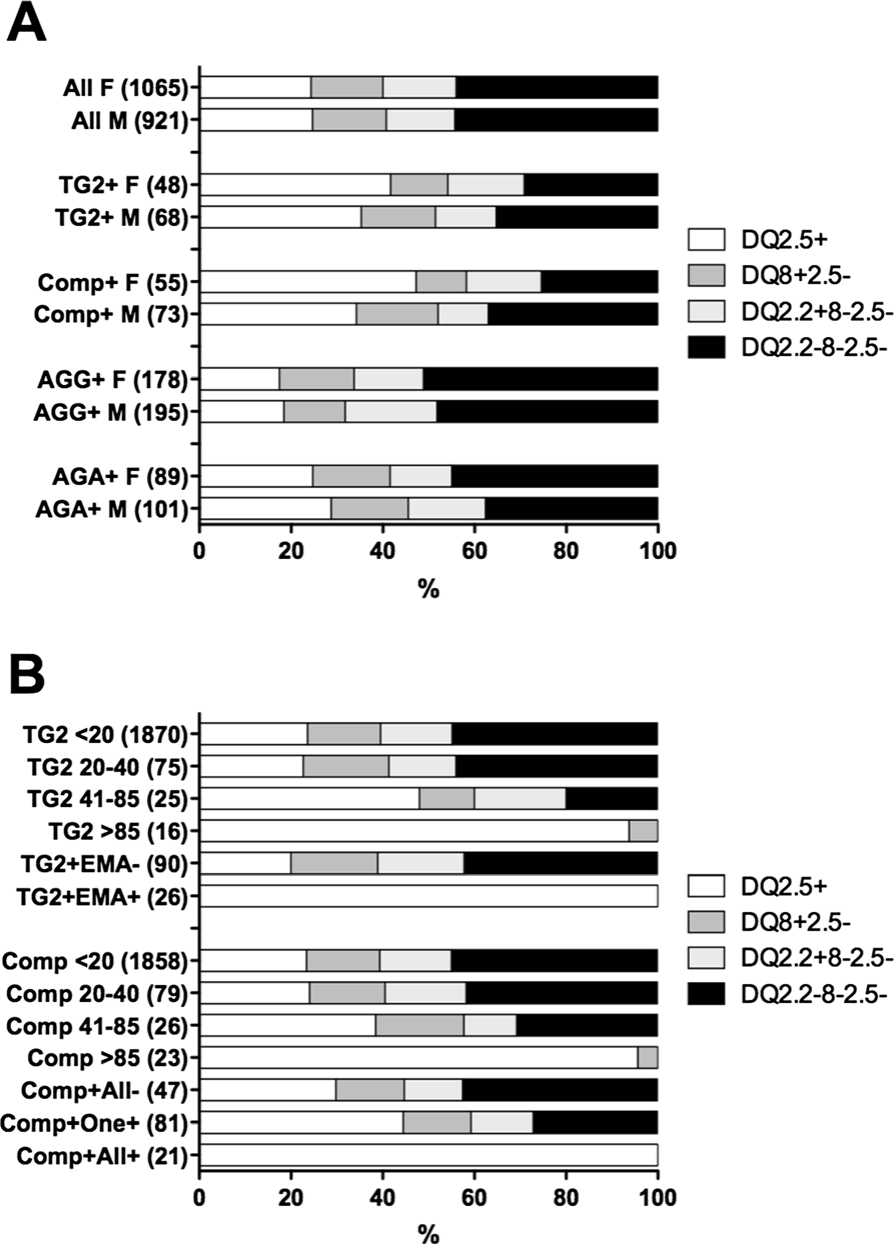
**Human leukocyte antigen (HLA-DQ genetic status and celiac disease (CD)-specific serology. (A)** HLA-DQ status of female (F) and male (M) community cohorts, and those who tested positive for anti-gliadin IgG (AGG) , anti-gliadin IgA (AGA), IgA specific for native human transglutaminase (TG)2, and composite TG2/DGP IgA/IgG (Comp.). Total numbers of subjects are in brackets. **(B)** HLA-DQ genotypes of women and men in the combined community cohorts classified by measured level of TG2 IgA and whether endomysial antibody (EMA) was also positive; or composite TG2/DGP IgA/IgG (Comp.) and whether all or at least one confirmatory serology (TG2 IgA, DGP IgA, or DGP IgG) was abnormal. Numbers of subjects are in brackets. DGP, Deamidated gliadin-derived peptide.

Abnormal TG2 IgA serology was found in 64 of 1,390 women (4.6%) and 80 of 1,158 men (6.9%). Although genetic susceptibility to CD was more common in seropositive women than in the community cohort (*P*<0.04) and showed a positive though non-significant trend in men, 29% of the women and 35% of the men who were seropositive for TG2 IgA were not genetically susceptible to CD (Figure 1A, Table 1). Higher levels of TG2 IgA were more strongly associated with HLA-DQ2.5, DQ8, and DQ2.2, and all 16 subjects with TG2 IgA levels above 85 U (4.5 × ULN) possessed HLA-DQ2.5 or HLA-DQ8 (Figure 1B). By contrast, genetic susceptibility to CD was the same in subjects with TG2 IgA levels of 20 to 40 U (53%) as it was in seronegative subjects (54%). Based on the absence of genetic susceptibility to CD in approximately one-third of subjects with sera showing increased levels TG2 IgA, it was clear that false-positive TG2 IgA findings are common in adults in the community. Hence, TG2 IgA serology alone would substantially overestimate the community prevalence of CD, and in clinical practice would be expected to result in many unnecessary gastroscopies to exclude CD.

**Figure 2:**
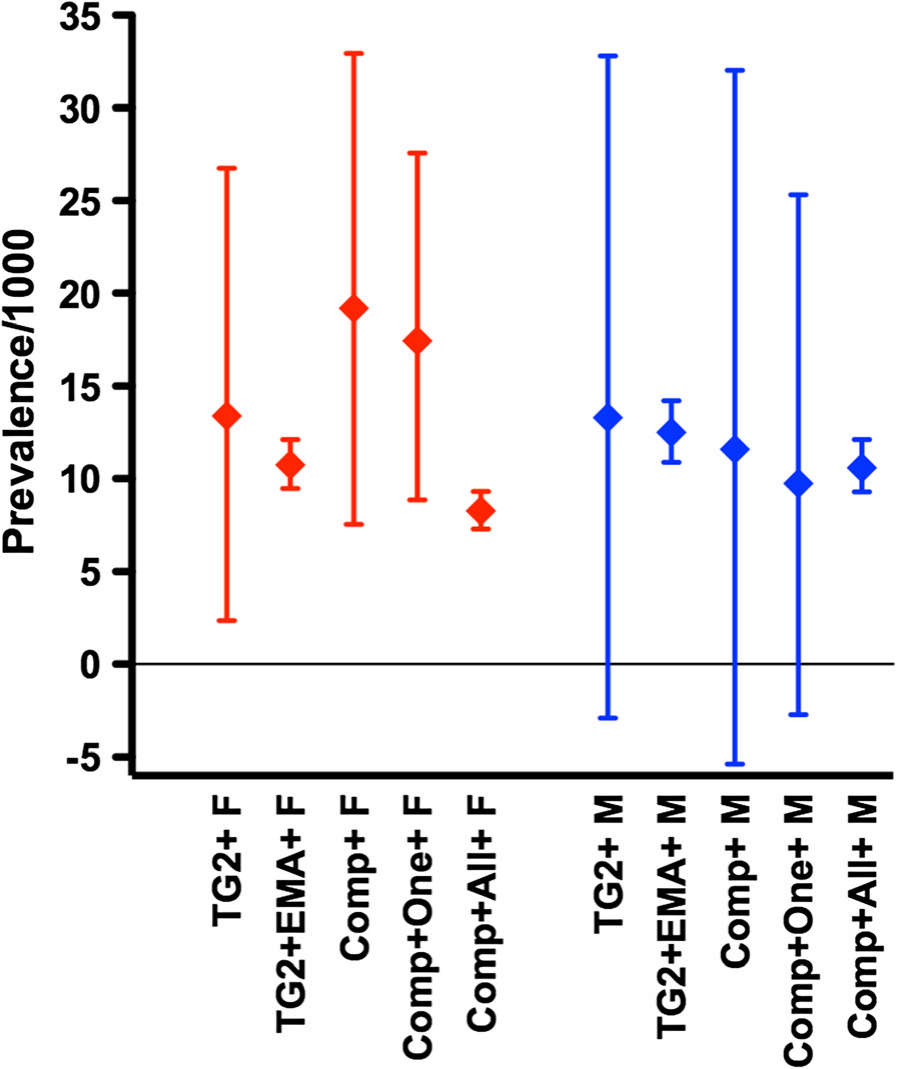
**Community prevalence estimates for celiac disease (CD).** Estimated prevalence of CD with 95% confidence intervals per 1,000 adult females (F) or male (M) subjects, based upon enrichment of human leukocyte antigen (HLA)-DQ2.5, DQ8, and DQ2.2 in subjects who were seropositive for transglutaminase (TG)2 IgA (TG2+), TG2 IgA+ and then endomysial antibody (EMA)+, composite TG2/DGP IgA/IgG (Comp+), Comp.+ and then TG2 IgA+, deamidated gliadin-derived peptide (DGP)-G+ or DGP-A+ (Comp + One+), and for Comp+ and then TG2 IgA+, DGP-G+ and DGP-A+ (Comp + All+).

These observations prompted us to formalize a simple method using contingency table analysis and RR to estimate the prevalence of CD using CD-associated serology and genetic susceptibility, assuming that all individuals with CD possessed HLA DQ2.5, DQ8, or DQ2.2 (see Statistical analysis). Subsequent analysis focused on 1,065 subjects from the female cohort and 921 from the male cohort with both serology and HLA-DQ status determinations. Based on this analysis, the observed enrichment for genetic susceptibility to CD in TG2 IgA-seropositive women and men could be accounted for if 14 of 48 seropositive women and 12 of 68 seropositive men had CD. These figures implied that only 14 of 34 women and 12 of 44 men with raised levels of TG2 IgA who possessed HLA-DQ2.5, DQ8, or DQ2.2 were actually affected by CD, and that the prevalence of CD was approximately 1.3% in both the female and male cohorts (Table 1; Figure 2).

To improve the precision of these prevalence estimates, ‘confirmatory’ EMA tests were performed and found to be positive in 13 women and 13 men with raised TG2 IgA levels. All 26 TG2 IgA+ EMA+ subjects possessed HLA DQ2.5 (*P*<0.0001). Hence, prevalence estimates for CD based on subjects being positive for both TG2 IgA and EMA were 1.1% (95% CI 1.0 to 1.2%) in women and 1.3% (95% CI 1.1 to 1.4%) in men (Table 1; Figure 1B; Figure 2).

### Expanded serological screening for celiac disease

It has been hypothesized that there are greater numbers of individuals in the community with genetically determined gluten-mediated disease than just those with raised levels of TG2 IgA or EMA [1,27], and some patients with biopsy-confirmed CD who are seronegative for TG2 IgA do have raised DGP IgA or IgG levels [28]. IgA and IgG anti-gliadin antibodies (AGA and AGG, respectively) have also been proposed as markers of gluten-responsive irritable bowel syndrome in patients genetically susceptible to CD. When tested in enrolment sera, both AGG and AGA were commonly raised in both the female (17% and 9%, respectively) and male (21% and 12%, respectively) cohorts, but genetic susceptibility to CD was not significantly increased in AGA or AGG seropositive women (55% and 49%, respectively) or men (62% and 52%) (Figure 1A).

The composite TG2/DGP IgG/IgA screen was abnormal in 78 of 1,390 women (5.6%) and 92 of 1,158 men (6.9%). Women with positive composite TG2/DGP IgG/IgA screen were more commonly positive for HLA-DQ2.5, DQ8, or DQ2.2 (75%, *P*<0.005) (Table 1, Figure 1). In men, over-representation of HLA-DQ2.5, DQ8, or DQ2.2 was not as pronounced and did not reach statistical significance (46/73; 63%). As observed with the TG2 IgA serology, higher readings in the composite TG2/DGP IgG/IgA screen were more strongly associated with genetic susceptibility to CD; all 23 subjects with levels above 85 U (4.5 × ULM) possessed HLA-DQ2.5 or HLA-DQ8 (Figure 1B). Based upon enrichment for HLA-DQ2.5, DQ8, or DQ2.2 in subjects with raised levels of composite TG2/DGP IgG/IgA, the prevalence of CD was 1.9% in women and 1.2% in men (Table 1; Figure 2). When subjects found to be seropositive by the composite TG2/DGP IgG/IgA screen were tested for TG2 IgA, DGP-G, and DGP-A separately, 65% (51/78) of women and 61% (56/92) of men had raised levels in at least one test. Prevalence estimates based on genetic enrichment and ‘confirmed’ abnormal TG2/DGP IgG/IgA screen were 1.8% in women and 1.0% in men (Table 1; Figure 2). All 10 women (0.9%) and 11 men (1.2%) with raised levels of each of the three serology tests (TG2 IgA, DGP-G, and DGP-A) were HLA-DQ2.5+ (*P*<0.0001) and also EMA+.

### Prospective review in seropositive cases

There was just one case of biopsy-confirmed CD at enrolment, a 57-year old male who was HLA-DQ2.5+ but negative in the TG2 IgA and composite TG2/DGP IgA/IgG screens, consistent with a diagnosis of CD but strictly following a gluten-free diet. Follow-up of the 51 women and 56 men having serum collected at enrolment who had ‘confirmed’ abnormalities after testing positive in the composite TG2/DGP IgG/IgA screen identified 10 women (0.7%) and 6 men (0.5%) with intestinal histology showing villous atrophy, crypt hyperplasia, and IELs, confirming active CD (Table 2; see Additional file 1: Tables S1A and S1B). Four of these women and two of these men had been diagnosed with CD between 2004 and 2009 on the basis of typical small bowel histology following investigation for diarrhea (n = 2), fatigue (n = 1), iron deficiency (n = 1), or osteoporosis (n = 1), or after diagnosis of a family member. The other biopsy-confirmed cases resulted from gastroscopies prompted by the current study in 2010. Two biopsy-confirmed cases were in women whose enrolment sera were negative for TG2 IgA, but positive for DGP-G and DGP-A. None of the cases identified with CD were deficient in IgA. Repeat serology after medical review supported CD in two further women (0.1%) and four additional men (0.3%), but these subjects were not investigated further, and the possibility of CD was rejected in two of these cases because the clinician judged that no symptoms suggestive of CD were present. Hence, 12 women (0.9%) and 11 men (0.9%) with abnormal serology at enrolment had the diagnosis of CD confirmed by histology or had repeat serology consistent with CD. In addition, three cases had equivocal changes on intestinal histology. In 9 women and 14 men, CD was excluded by serology, HLA-DQ genetic testing, or histology findings. Medical review was not possible for 29 women and 23 men, and in 6 subjects, medical follow-up led to exclusion of CD without further relevant investigations.

**Table 2:** **Follow-up and final disposition of subjects**^**a**^

	**Women, n**	**Men, n**
Total subjects	1,390	1,158
CD diagnosed by histology before enrolment	0	1
Abnormal composite TG2/DGP IgA/IgG, and TG2 IgA, or DGP IgA or IgG	51	56
Findings diagnostic/supportive of untreated CD		
Histological diagnosis: Intestinal villous atrophy, crypt hyperplasia, and IELs		
Prompted by current study, 2010	6	4
During standard medical care between 2004 and 2009	4	2
Serological diagnosis: confirmation of multiple CD serological abnormalities		
No supporting histological evidence obtained	2	3
Treating doctor excluded CD because patient was asymptomatic	0	2
Findings equivocal for CD		
Intestinal IELs +/− mild focal villous atrophy, or villous atrophy and crypt hyperplasia without IELs	0	3
Findings excluded/were not supportive of CD		
Normal intestinal histology without serological testing	2	5
Serological exclusion: CD serological abnormalities not replicated	7	8
Genotyping exclusion: testing for HLA DQ2.5/8/2.2 negative	0	1
Follow-up not possible or not undertaken		
Treating doctor did not investigate further as subject asymptomatic and/or performed blood tests unrelated to CD	1	5
Subject deceased and CD not diagnosed pre-mortem	8	5
Subject declined follow-up medical review	16	11
Subject could not be contacted; lost to follow-up	5	7
CD cases estimated by serogenetic modeling, range	12 to 26	12 to 16
Lower 95% CI for CD cases based on TG2+ EMA+	11	12

Abbreviations: CD, celiac disease; DGP, Deamidated gliadin-derived peptide; EMA, endomysial antibody; HLA, human leukocyte antigen; IEL, Intra-epithelial lymphocyte; Ig, immunoglobulin; TG, transglutaminase.

^a^Data are n, unless otherwise stated.

Sera collected from 597 women at the 10-year review showed that 24 (4.0%) had persistent increases in the composite TG2/DGP IgA/IgG and/or TG2 IgA screen; 22 of these subjects had known genotype, and of these, 21 (95%) were genetically susceptible to CD (see Additional file 2: Table S2). At the 10-year review, serological abnormalities persisted in all genetically susceptible subjects whose enrolment sera had showed a composite TG2/DGP IgA/IgG result of 59 U or higher (n = 13) or TG2 IgA of 58 U or higher (n = 11). Less pronounced increases in the composite TG2/DGP IgA/IgG and TG2 IgA screens at enrolment were less often reproduced at the 10-year review. There were three cases showing abnormalities in both composite TG2/DGP IgA/IgG and TG2 IgA screens at 10-year follow-up that were not present at enrolment, in two cases EMA was reported as negative and in one it was equivocal (see Additional file 2: Table S2). Both of the genotyped cases showing new serological abnormalities at 10-year follow-up were genetically susceptible to CD. Intestinal histology was not available for these three patients.

### Diagnostic pathways

Prompted by the observed high rate of false-positive serology tests, we designed a series of diagnostic algorithms using either TG2 IgA or composite TG2/DGP IgA/IgG screens or HLA-DQ genotyping as the initial screen, with or without subsequent confirmatory serology and/or HLA-DQ genotyping prior to gastroscopy and definitive small bowel histology (Table 3). The overall cost and resource utilization of models were compared using the serology and genotyping datasets generated from the female and male cohorts. We assumed that the total numbers of affected cases were those predicted by genetic enrichment in TG2 IgA or composite TG2/DGP IgA/IgG screen-positive subjects (13/1,000 women and 13/1,000 men, and 19/1,000 women and 12/1,000 men, respectively) (Table 4). Because many cases now tested for CD in Australia occur in the setting of primary care, where the pre-test probability of CD is unlikely to be substantially different from background community prevalence, this analysis may be relevant to community clinical practice.

**Table 3:** Models for initial and confirmatory testing of CD

**Model**	**Initial test**	**Additional tests if initial test positive**	**Definitive test**
A	TG2 IgA	None	Biopsy
B	TG2 IgA	HLA-DQ	Biopsy if HLA-DQ2.5/2.2/8+
C	TG2 IgA	EMA and HLA-DQ	Biopsy if HLA-DQ2.5/2.2/8+
D	TG2 IgA	If TG2 IgA > 10 × ULN, then EMA and HLA-DQ; otherwise none	None if TG2 IgA > 10 × ULN, EMA abnormal and HLA-DQ2.5/2.2/8+; otherwise biopsy
E	HLA-DQ	TG2 IgA; if TG2 IgA 1 to 3 × ULN, then EMA	Biopsy if TG2 IgA >3 × ULN or EMA abnormal
F	Composite TG2/DGP IgA/IgG	None	Biopsy
G	Composite TG2/DGP IgA/IgG	HLA-DQ	Biopsy
H	Composite TG2/DGP IgA/IgG	HLA-DQ, TG2 IgA, DGP-G and DGP-A	Biopsy if HLA-DQ2.5/2.2/8+ and either TG2 IgA or DGP-G or DGP-A abnormal

Abbreviations: DGP, Deamidated gliadin-derived peptide; EMA, endomysial antibody; HLA, human leukocyte antigen; TG, transglutaminase; ULN, upper limit of normal.

**Table 4:** **Models of diagnosis:**^**a**^**overall cost**^**b**^**and numbers of investigations for screening 1,000 adult men or women in the community for CD**

**Screen**	**Sex**	**Model**							
		**A**	**B**	**C**	**D**	**E**	**F**	**G**	**H**
TG2 IgA^c^	F	1,000	1,000	1,000	1,000	–	–	–	–
	M	1,000	1,000	1,000	1,000	–	–	–	–
Composite TG2/DGP IgA/IgG^c^	F	–	–	–	–	–	1,000	1,000	1,000
	M	–	–	–	–	–	1,000	1,000	1,000
HLA-DQ^d^	F	–	–	–	–	1,000	–	–	–
	M	–	–	–	–	1,000	–	–	–
Confirmatory test if initial test is ‘positive’									
TG2 IgA^c^	F	–	–	–	–	560	–	–	–
	M	–	–	–	–	557	–	–	–
EMA^c^	F	–	–	45	3	19	–	–	–
	M	–	–	69	2	37	–	–	–
TG2 IgA and DGP-G, DGP-A^e^	F	–	–	–	–	–	–	–	52
	M	–	–	–	–	–	–	–	79
HLA-DQ^d^	F	–	45	45	3	–	–	52	52
	M	–	69	69	2	–	–	79	79
Biopsy if initial and/or confirmatory tests are positive^f^									
Biopsy	F	45	32	32	42	18	52	38	28
	M	69	45	45	67	16	79	48	24
Cases diagnosed^g^	F	13	13	13	13	13	19	19	19
	M	13	13	13	13	13	12	12	12
Total cost for investigations, A$	F	63,150	57,484	58,605	61,034	149,367	69,100	63,422	57,010
	M	83,550	71,406	73,124	82,139	148,041	92,050	75,152	57,924
Cost per case screened, A$	F	63	57	59	61	149	69	63	57
	M	84	71	73	82	148	92	75	58
Cost per case diagnosed, A$	F	4,858	4,422	4,508	4,695	11,490	3,637	3,338	3,001
	M	6,427	5,493	5,625	6,318	11,388	7,671	6,263	4,827

Abbreviations: CD, celiac disease; DGP, Deamidated gliadin-derived peptide; EMA, endomysial antibody; HLA, human leukocyte antigen; TG, transglutaminase.

^a^Data are n, unless otherwise stated.

^b^Costs are based on 100% reimbursement of Australian Medicare Benefits Schedule fee for items ^c^71163: A$24.90, ^d^71151: A$119.65, ^e^71164: A$40.15, and ^f^A$850 estimated costs of gastroscopy, histology, sedation, and hospital/clinic facility fee.

^g^Cases diagnosed were assumed to be the excess of HLA-DQ2.5/8/2.2 subjects in those screened by TG2 IgA (models A to E), or composite antigen ELISA (model F to H), as detailed in Table 1.

Model A (initial testing for TG2 IgA followed by small bowel biopsy) is widely recommended in clinical guidelines, but according to the Medicare Australia item costs, is more expensive and results in more (unnecessary) gastroscopies than the model (model B) including HLA-DQ genotyping to exclude ‘gene-negative’ TG2 IgA false-positive patients prior to biopsy. Including both confirmatory EMA and HLA-DQ genotyping for TG2 IgA-seropositive patients, but still performing gastroscopies in all of this patient group (model C) modestly increases cost and has the advantage that cases with a pre-biopsy likelihood of CD greater than 90% can be identified to minimize the risk of biopsy false-negatives. Model D is based on that proposed by the revised ESPGHAN 2012 criteria for symptomatic children, avoiding the requirement for biopsy in patients with TG IgA levels greater than 10 × ULN who have confirmatory EMA and HLA-DQ genotyping. Only 0.2% of men and 0.3% of women in the community would be expected to have levels of TG2 IgA above 10 × ULN, and model D was little different from model A in its cost and clinical impact. Model E is based on that proposed by the revised ESPGHAN 2012 criteria for asymptomatic children at increased risk; the initial test is HLA-DQ genotyping followed by biopsy if TG2 IgA is greater than 3 × ULN, or if TG2 IgA is raised but is less than 3 × ULN then confirmatory EMA is performed, and if positive, then biopsy is undertaken. The cost associated with model E is about double that of the next most expensive model, but requires the least number of gastroscopies. Model E is the same as model A except that the composite TG2/DGP IgA/IgG screen is substituted for TG2 IgA; it is cheaper than model A per case diagnosed as it detects additional affected female patients who might be TG2 IgA-seronegative. Including ‘confirmatory’ HLA-DQ genotyping also reduces the cost of initial testing with the composite TG2/DGP IgA/IgG screen (model G), as it did for TG2 IgA screening in model B. Finally, in model H, biopsies are performed only in patient with positive results on composite TG2/DGP IgA/IgG screen who are confirmed to be genetically susceptible to CD and who also show abnormalities in confirmatory TG2 IgA, DGP-G or DGP-A tests. Model H reduced the cost per case diagnosed by 38% in women and 25% in men, and reduced the number of gastroscopies by 38% in women and 65% in men.

## Discussion

The concept of a ‘celiac iceberg’ has been important in drawing attention to a large, unrecognized group of patients with CD who do report symptoms considered ‘typical’ of CD [29]. Investigators have proposed expansion of the ‘iceberg’ to encompass patients who are genetically susceptible to CD, but show only raised IEL counts or an isolated abnormal CD-specific serology and normal intestinal histology [30-32]. Consequently, there is considerable uncertainty regarding the true extent of gluten-mediated disease in the community. The recent revision of diagnostic criteria proposed by the ESPGHAN Working Group seeks to address these developments, and increase the contribution of serology and HLA-DQ genetic tests to diagnosis [33-36].

However, Walker *et al*. highlighted the difficulties in defining the community prevalence of CD [1]. From 1,000 randomly selected Swedish adults who underwent diagnostic gastroscopy, 33 were TG2 IgA-positive, and 16 of these were also EMA-positive. These 16 subjects who were seropositive for both TG2 IgA and EMA were considered to have CD, but only 12 of the 16 EMA subjects showed both villous atrophy and increased density of IELs; of the other 4 patients, 3 showed only increased IELs and the fourth had normal histology. In addition, two of the TG2 IgA+ EMA− subjects also showed partial villous atrophy and raised IEL numbers. Walker *et al*. speculated that if increased TG2 IgA with normal intestinal histology or raised IEL numbers without increased TG2-IgA reflected gluten-mediated pathology, then the community prevalence of CD might be considerably greater than their estimate of almost 2% [1].

Consistent with a previous study from Australia and several from Europe and North America [14,20,37,38], over 98% of locally recruited volunteers with ‘biopsy-confirmed’ CD possessed HLA-DQ2.5, DQ8, or DQ2.2. In addition, we found that absence of HLA-DQ2.5, DQ8, or DQ2.2 in ‘biopsy-confirmed’ cases predicted incorrect diagnosis. Building upon this observation, we developed a novel method to estimate the prevalence of CD in two large age-stratified, randomly selected community cohorts, based on the relative enrichment for HLA-DQ alleles conveying genetic susceptibility to CD in the group of individuals with abnormal CD-specific serology tests. If, as some previous studies have done, we had assumed that all TG2 IgA-seropositive individuals with HLA-DQ2.5, DQ8, or DQ2.2 did have CD, then we would have estimated community prevalence to be 3.1% in women and 4.8% in men. The novel serogenetic approach we developed potentially overcomes not only the need for biopsy but also performance variation between different TG2 IgA ELISAs when estimating the prevalence of CD in the community. In this study, we estimated that 1.3% of men and women in the community had unrecognized CD using TG2 IgA serology. However, our finding that two TG2 IgA-seronegative women who were positive in the composite TG2/DGP IgA/IgG screen were subsequently confirmed to have CD on small bowel histology added support to the higher prevalence figure of 1.9% for women, estimated using the composite TG2/DGP IgA/IgG screen.

Clinical follow-up confirmed 10 new cases of unrecognized CD in addition to 7 cases diagnosed through standard medical care before or during the current prospective study. As anticipated, the biopsy-confirmed prevalence of CD of 0.7% in women and 0.6% in men was substantially less than by the serogenetic method. However, for a variety of reasons, seven additional cases with persistent serological abnormalities did not undergo gastroscopy, and more than half of the cases who were positive in the composite TG2/DGP IgA/IgG screen were not available for follow-up or had no additional investigation. Medical review of seropositive subjects by their primary care physicians also highlighted the persisting misconception that CD only presents with classical symptoms. Although use of a gluten-free diet has become much more popular since the enrolment sera were collected, none of the subjects with raised CD-specific serology reported having adopted gluten-free diet, apart from those actually diagnosed with CD. However, it is possible that deliberate or inadvertent reduction of dietary gluten by some formerly seropositive subjects could have resulted in normalization of serological abnormalities.

Testing for CD is increasing. Claims for serological testing for CD reimbursed by Medicare Australia in the State of Victoria rose from 9.3 per 1,000 population in 2004 to 14.4 per 1,000 population in 2009; the total number of claims during this 6-year period was 72.4 per 1,000 population. Females and males aged over 15 years accounted for 63% and 23%, respectively, of all Medicare claims for coeliac disease serology tests in 2010. Between 2004 and 2009, 4 of the estimated 12 to 26 unrecognized cases of CD in our female cohort and 2 of the estimated 12 to 16 unrecognized cases in our male cohort were diagnosed through standard medical care. Over time, as testing increases, the total number of undiagnosed adult cases of CD would be expected to fall steadily, particularly in women. The combined effects of increased testing and increased diagnoses would eventually reduce the pre-test probability of CD when testing patients in primary care. TG2 IgA serology is widely recommended as the initial investigation when CD is considered, but based on serogenetic data from the current study, fewer than one in three women and one in five men with abnormal TG2 IgA in the community would be expected to have unrecognized CD. Hopper *et al*. also reported similarly high rates of false positives using a different TG2 IgA assay in a group of 2,000 patients referred for gastroscopy [8]. If gastroscopy with intestinal biopsy continues to be recommended as the next step after positive TG2 IgA serology, many unnecessary gastroscopies will be performed, at considerable expense and inconvenience for patients.

To address this problem, we modeled the practical and financial effects of including confirmatory serology and genetic testing after initial testing and before ‘definitive’ small bowel histology. Inclusion of genetic testing after either TG2 IgA or composite TG2/DGP IgA/IgG serology reduced gastroscopies and overall costs incurred according to the current Medicare Australia reimbursement schedule. Inclusion of confirmatory serology after initial testing with composite TG2/DGP IgA/IgG serology had a similar effect by reducing gastroscopies without appreciably affecting detection rates, and also lowering overall costs according to pricings in Australia. The applicability and financial effect of the diagnostic algorithms are likely to vary between countries and healthcare systems, depending on local laboratory facilities and costing. However, the community rates of ‘positive’ and ‘negative’ serologies and the genetic susceptibility data presented in the current study can be combined with relevant local costs for laboratory assays and procedures to predict which diagnostic pathway might be the most relevant and cost-effective in a particular setting.

Although it would be premature to formally propose a ‘single’ diagnostic flow chart, the present study highlights the effects of separating initial laboratory testing by serology from a second set of laboratory tests in order to further increase the likelihood of CD before proceeding to endoscopic biopsy and histology, which is definitive but also the most expensive and intrusive investigation used in diagnostic investigations for CD. Shifting costs from gastroscopy to laboratory testing promises to reduce overall costs and may be more acceptable to patients, particularly if gastroscopy is difficult to access or even undesirable.

## Conclusions

The exceptional performance characteristics of CD-specific serology and HLA-DQ genetic testing have prompted the release of new serogenetic criteria to diagnose CD. In the current study, a novel serogenetic methodology was developed to formally estimate the community prevalence of CD by optimizing combined serological and genetic testing. In contrast to conflicting reports that the prevalence of CD is overestimated or underestimated due to discordance in intestinal histology and TG2-IgA serology, screening using TG2-IgA, EMA, and HLA-DQ genetic testing indicates that the prevalence of CD in adults is at least 1.1% in men and 1.0% in women, but when TG2 and DGP serology are combined as initial screening tools, sensitivity is improved, and the estimated prevalence of CD is 1.2% or 1:86 in men and 1.9% or 1:52 in women. Serogenetic screening without the requirement for follow-up small bowel biopsies provides a flexible, cost-effective methodology that could be widely applied to obtain accurate estimates of the prevalence of CD in large cohort studies.

In the current study, the serogenetic screening also highlighted that false-positive TG2-IgA serology is common in adults at average risk of CD. Using serology and genetic tests after initial serology screening is both cost-effective and could avoid many unnecessary gastroscopies prompted by abnormal ‘CD-specific’ serology.

## Abbreviations

AGA: Anti-gliadin IgA; AGG: Anti-gliadin IgG; CD: Celiac disease; CI: Confidence interval; DGP: Deamidated gliadin-derived peptides; DGP-A: Deamidated gliadin-derived peptides-immunoglobulin A; DGP-G: Deamidated gliadin-derived peptides-immunoglobulin G; ELISA: Enzyme-linked immunosorbent assay; EMA: Endomysial antibody; ESPGHAN: European Society for Pediatric Gastroenterology, Hepatology, and Nutrition; HLA: Human leukocyte antigen; IEL: Intra-epithelial lymphocyte; Ig: Immunoglobulin; PCR: Polymerase chain reaction; RR: Relative risk; SNP: Single-nucleotide polymorphism; SSO: Sequence-specific oligonucleotide; TG: Transglutaminase; ULN: Upper limit of normal.

## Competing interests

RPA is Chief Scientific Officer of ImmusanT Inc., and shareholder in ImmusanT, Inc. and Nexpep Pty Ltd, companies that are directly or indirectly involved in developing treatments and diagnostics for CD. RPA has been a consultant for INOVA Diagnostics, Prometheus Laboratories, and Given Imaging (USA), companies that provide diagnostics for CD. JT-D is a shareholder in Nexpep Pty Ltd., and a consultant to ImmusanT, Inc. RPA and JTD are named inventors on patents related to diagnosis and treatment of CD. The Walter and Eliza Hall Institute of Medical Research hold shares in Nexpep Pty Ltd.

## Authors’ contributions

RPA participated in study concept and design, overall supervision, data gathering and analysis, and manuscript preparation; MJH in study design, sample management, data gathering, and manuscript revision; RT in serology assays and manuscript revision; PD in genotyping, data interpretation, and manuscript revision; ELD in study design, genotyping, data interpretation, and manuscript revision; MJC in sample management, data gathering and manuscript revision; KA in sample preparation, and manuscript revision; GCN in study design, supervision, and revision of the manuscript; JTD in study design, data interpretation, and manuscript revision; REK in expert clinical advice and follow-up, and revision of the manuscript; WP and BHT in study design and revision of the manuscript; MAK and MAB in study design, supervision, and revision of the manuscript; and JAP in study design, sample management, data gathering, and manuscript revision. All authors read and approved the final manuscript.

## Pre-publication history

The pre-publication history for this paper can be accessed here:

http://www.biomedcentral.com/1741-7015/11/188/prepub

## Supplementary Material

Additional file 1: Tables S1 (A) Baseline serogenetic findings and follow-up in females with confirmed abnormal transglutaminase 2/deamidated gliadin peptide (TG2/DGP) lgA/lgG when recruited 1994 to 1997. (B) Baseline serogenetic findings and follow-up in men with confirmed abnormal composite TG2/DGP lgA/lgG at recruitment during the period 2001 to 2006. Click here for file

Additional file 2: Table S2 Persistent or reproducible abnormal serology in 597 women at baseline and 10 years later. Click here for file
