# Supplementary figures and images for: A novel serogenetic approach determines the community prevalence of celiac disease and informs improved diagnostic pathways
Source: BMC Med. 2013 Aug 28;11:188. doi: 10.1186/1741-7015-11-188 (PMC3765645; doi:10.1186/1741-7015-11-188)

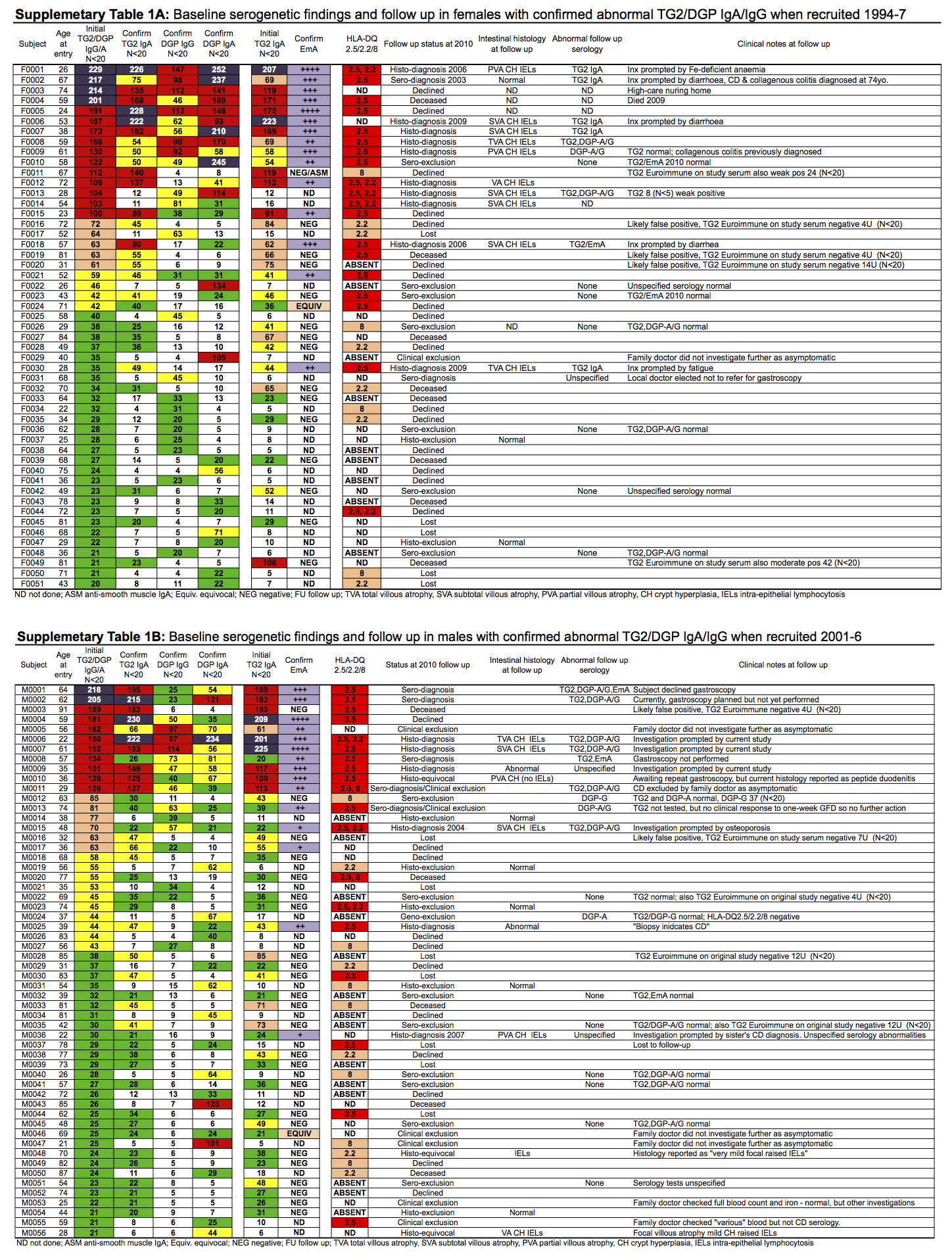

Supplement: Additional file 1: Tables S1 — (A) Baseline serogenetic findings and follow-up in females with confirmed abnormal transglutaminase 2/deamidated gliadin peptide (TG2/DGP) lgA/lgG when recruited 1994 to 1997. (B) Baseline serogenetic findings and follow-up in men with confirmed abnormal composite TG2/DGP lgA/lgG at recruitment during the period 2001 to 2006. [file 1741-7015-11-188-S1.tiff]

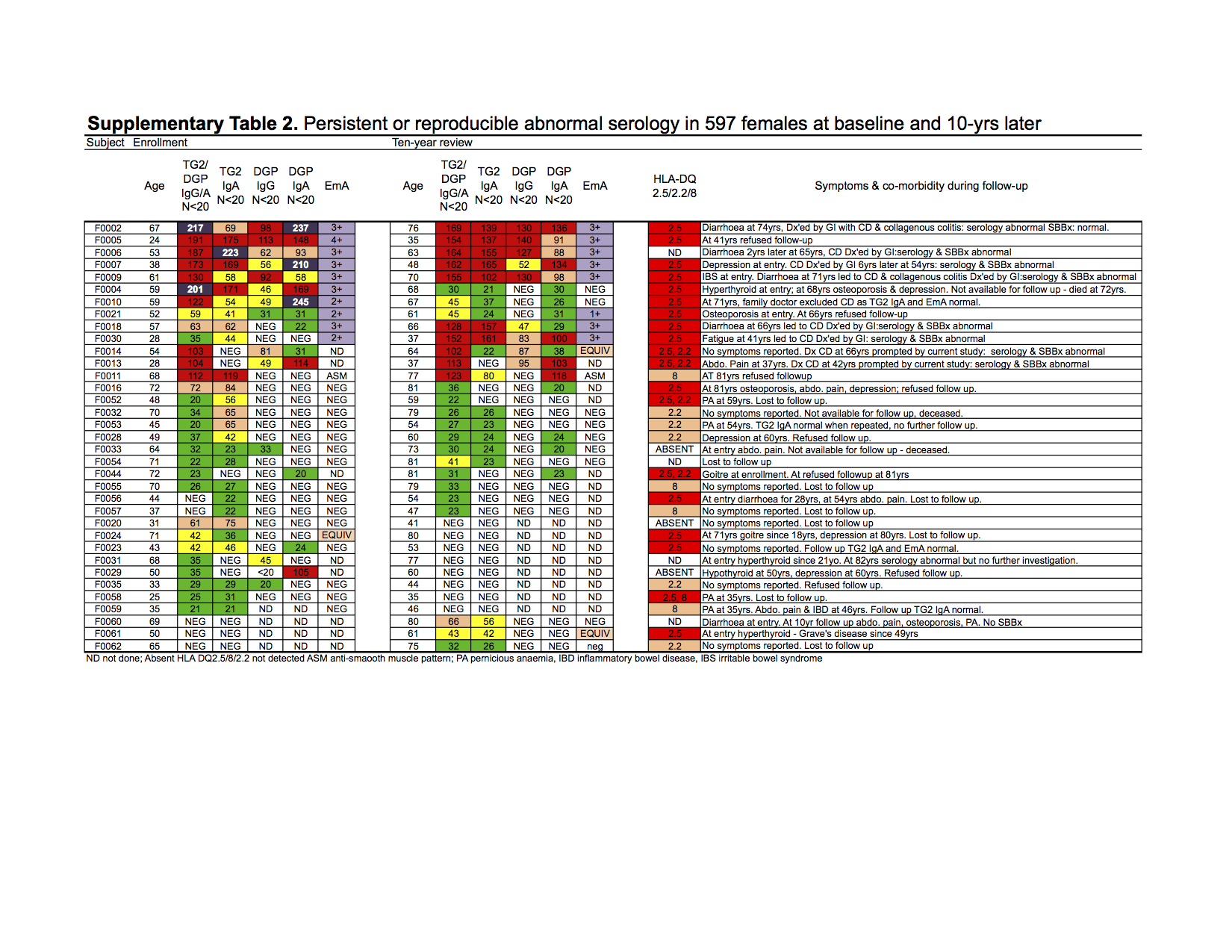

Supplement: Additional file 2: Table S2 — Persistent or reproducible abnormal serology in 597 women at baseline and 10 years later. [file 1741-7015-11-188-S2.tiff]
